# Assessing vitamin E acetate as a proxy for E-cigarette additives in a realistic pulmonary surfactant model

**DOI:** 10.1038/s41598-024-75301-8

**Published:** 2024-10-11

**Authors:** Hanna Korolainen, Agnieszka Olżyńska, Wojciech Pajerski, Paulina Chytrosz-Wrobel, Ilpo Vattulainen, Waldemar Kulig, Lukasz Cwiklik

**Affiliations:** 1 Department of Physics, University of Helsinki, P.O. Box 64, 00014 Helsinki, Finland; 2 J. Heyrovský Institute of Physical Chemistry of the Czech Academy of Sciences, Dolejškova 2155/3, 182 23 Prague, Czech Republic; 3 InnoRenew CoE, Livade 6a, 6310 Izola, Slovenia; 4 Institute of Organic Chemistry and Biochemistry, Czech Academy of Sciences, Flemingovo nám. 542/2, 160 00 Prague, Czech Republic

**Keywords:** Pulmonary surfactant, Lung surfactant, Vaping-associated pulmonary injury, EVALI, Molecular dynamics simulation, Biophysical chemistry, Computational biophysics, Membrane biophysics

## Abstract

Additives in vaping products, such as flavors, preservatives, or thickening agents, are commonly used to enhance user experience. Among these, Vitamin E acetate (VEA) was initially thought to be harmless but has been implicated as the primary cause of e-cigarette or vaping product use-associated lung injury, a serious lung disease. In our study, VEA serves as a proxy for other e-cigarette additives. To explore its harmful effects, we developed an exposure system to subject a pulmonary surfactant (PSurf) model to VEA-rich vapor. Through detailed analysis and atomic-level simulations, we found that VEA tends to cluster into aggregates on the PSurf surface, inducing deformations and weakening its essential elastic properties, critical for respiratory cycle function. Apart from VEA, our experiments also indicate that propylene glycol and vegetable glycerin, widely used in e-liquid mixtures, or their thermal decomposition products, alter surfactant properties. This research provides molecular-level insights into the detrimental impacts of vaping product additives on lung health.

## Introduction

Electronic nicotine delivery systems (ENDS), known as e-cigarettes, have experienced a surge in popularity over the past two decades since they were first introduced to the public in the early 2000s. The market of ENDS has gone through first generation pilot products, second generation ‘vape pens’, third generation ‘mods’, ending up with fourth generation ‘pods’ with new technology^1,2^. E-cigarettes consist of a battery, an atomizer with a heating coil to vaporize the e-liquid and create an aerosol, an e-liquid chamber, and a mouthpiece. The e-liquid typically contains nicotine, a base solution which is a mixture of propylene glycol (PG) and vegetable glycerin (VG), and optionally flavors^2–4^. The heating process during vaping results in thermal degradation of e-liquid components, resulting in the formation of toxic compounds such as formaldehyde, acetaldehyde, acrolein, propanol, benzaldehyde, carbon monoxide, nitrosamines, and free radicals^3–5^. Not surprisingly, inhaling e-cigarette vapors can have toxic effects on the lungs and adverse effects on the cardiovascular system as well as affect other tissues^2,6–8^.

However, the most significant acute problem of vaping is EVALI (e-cigarette or vaping product use-associated lung injury), which is a lung disease whose symptoms include shortness of breath, cough, and chest pain. Although this disease has emerged very recently, it has still proven to be serious. For example, the EVALI epidemic that broke out in the United States in 2019 resulted in nearly 2,600 hospitalized patients and 60 fatal cases^9,10^. Most of the reported cases of EVALI were related to vaping products containing tetrahydrocannabinol (THC). Analysis of bronchoalveolar lavage samples from these EVALI patients identified vitamin E acetate (VEA), which was used in relatively high concentrations as a thickening agent in THC-containing products. A follow-up study of EVALI patients revealed the presence of VEA in 94% of cases^11^, confirming previous findings and emphasizing the role of VEA in the pathophysiology of EVALI^12^. It has been suggested that when inhaled, VEA partitions into the pulmonary surfactant, altering its normal function^13–16^. Apart from VEA itself, its thermal decomposition products have also been found to contribute to the development of EVALI^17–20^.

Pulmonary surfactant (PSurf) is a complex membrane layer formed by lipids and proteins, covering the walls of the lung alveoli, where gas exchange takes place, especially with regard to oxygen and carbon dioxide. PSurf creates an interface between air and liquid in the lungs, reducing the surface tension of the alveoli, preventing their collapse during breathing, and forming a barrier against inhaled allergens and/or pathogens^21,22^. Among e-cigarette users, disruption of PSurf homeostasis leads to the development of respiratory distress^10,13^.

Even though VEA found in vaping liquids has been identified as a trigger for EVALI^11,12,23^, the exact interaction mechanism between VEA and other critical components of the lungs, especially PSurf, is still unclear. The main reason for this is that the experimental study of PSurf is exceptionally difficult because its structure is not known well enough, and the problem is not eased by the fact that PSurf is in a constant state of change during the respiratory cycle. To elucidate the mechanism of lung dysfunction caused by EVALI, the development of new research methods is therefore crucial. Since the EVALI outbreak linked to high concentrations of VEA, its usage in vaping products has been eliminated. However, VEA remains a critical example of how additives perceived as harmless, such as colorants or flavoring found at ratios up to 10%, can prove to be extremely detrimental when included in vaping products. This incident underscores the need for thorough evaluation and regulation of substances added to e-cigarettes.

Recent experiments have shown that VEA prevents lung surfactant membranes from reaching high surface pressure, and this effect has been hypothesized to be due to interactions of VEA with lipids and pulmonary proteins^14^. Furthermore, biomolecular simulations have been used to investigate the properties of lipid membranes^24–26^, and these computational studies have explored the effects of e-cigarette vapor components such as nicotine (and its derivatives)^27,28^, propylene glycol^29^, and menthol^27^. Qin et al.^30,31^ used molecular dynamics (MD) simulations to study the position of α-tocopherol in different phospholipid bilayers. They found that the hydroxyl group of α-tocopherol was located at approximately the same depth as the center of the DMPC *sn*-2 chain. Leng et al.^32,33^ performed both MD simulations and neutron scattering experiments to investigate the location of α-tocopherol molecules in polyunsaturated and monounsaturated lipid bilayers. They discovered that α-tocopherol has less affinity for polyunsaturated lipids in these systems. Boonnoy et al.^34^ investigated the flip-flop frequency of tocopherol molecules in oxidized and unoxidized lipid bilayers. Their results showed that the flip-flop frequency was significantly higher in oxidized bilayers than in unoxidized ones, supporting the view of tocopherol as an antioxidant. Overall, these and previous studies^35–38^ have focused on understanding the location, orientation, and flip-flop of tocopherol molecules in lipid layers in connection with the protective role of tocopherol against free radicals, but have not elucidated the mechanism by which VEA inhibits or weakens the function of PSurf.

In this study, we used VEA as a proxy for e-cigarette additives and we employed several experimental techniques together with atomic-level MD simulations to investigate the behavior of PSurf models when exposed to VEA. To render this possible, and to monitor the effects of PSurf surface pressure changes during vaporization, we developed an e-cigarette vapor exposure system, which we used as the main technique to identify changes in PSurf in vitro. Further, the Langmuir trough was used to evaluate the stability of the film and to investigate its heterogeneity at the microscale. These experimental techniques were complemented by in silico atomistic MD simulations used to elucidate at the molecular level how VEA weakens the properties of PSurf. The results inform that the mechanism of action of VEA is based on its tendency to cluster into aggregates on the PSurf surface, induce deformations in the structure of PSurf, and thereby weaken the PSurf’s elastic properties, which are essential for the function of PSurf during the respiratory cycle.

## Materials and methods

### Materials

Poractant alfa (Curosurf), an extract of natural porcine lung surfactant produced by Chiesi Farmaceutici (Parma, Italy) was used. 1,2-dipalmitoyl-*sn*-glycero-3-phosphocholine (DPPC), 1-palmitoyl-2-oleyl-*sn*-glycero-3-phosphocholine (POPC), 1-palmitoyl-2-oleoyl-*sn*-glycero-3-phospho-(1’-rac-glycerol) (POPG), and cholesterol (ovine wool) (Chol) were ordered from Avanti Polar Lipids (Alabaster, AL). The fluorescent probe 1,2-dioleoyl-*sn*-glycero-3-phosphoethanolamine-Atto633 (DOPE–Atto633) was supplied by ATTO-TEC (Siegen, Germany). Propylene glycol (PG), vegetable glycerin (VG), α-tocopherol (vitamin E) acetate (VEA), and ethylenediaminetetraacetic acid (EDTA) were purchased from Sigma-Aldrich (St. Louis, MO). Phosphate buffered saline (PBS) and chloroform of spectroscopic grade were ordered from Merck (Darmstadt, Germany). The buffer was prepared using Milli-Q water (Millipore, USA). All chemicals were used without further purification.

### e-Cigarette vapor exposure system

**Fig. 1:**
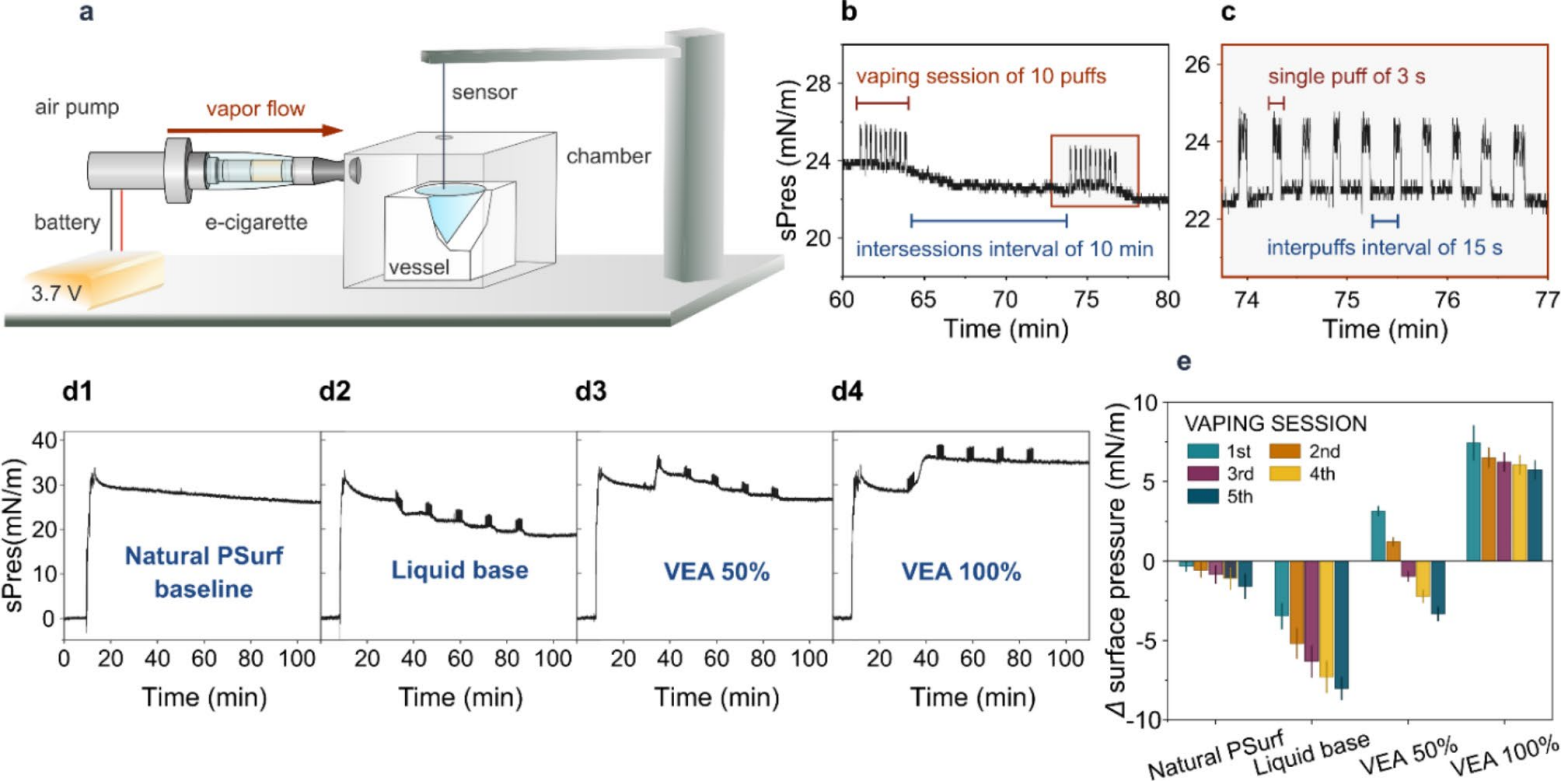
Exposure of the natural PSurf to e-cigarette vapor. **a** Schematic diagram of the e-cigarette vapor exposure system. An electric air pump was used to blow the e-cigarette vapor into the measurement chamber with the vessel containing the natural PSurf film. A sensor probe for measuring surface pressure was inserted through an opening in the lid of the chamber. **b** The vaping protocol consisted of 5 vaping sessions 10 min apart. **c** Each vaping session contained 10 puffs of 3 s with a 15 s interval between puffs. **d1** Control measurement of surface pressure (sPres) for natural PSurf, where the system is stabilized during the first 8 min after which the PSurf film is added (between 8 and 9 min), followed by a second stabilization phase for ~ 20 min, and the actual measurement starts at 30 min. In this control measurement, e-cigarette vapor is not added. **d2** PSurf exposed to the e-liquid base solution, where the exposure is initiated at 30 min. **d3** PSurf exposed to 50% vitamin E acetate (VEA) in a base solution. **d4** PSurf exposed to a pure VEA solution. **e** Changes in the surface pressure of pulmonary surfactant model corresponding with representative curves depicted in panels **d1**–**d4**

The experiments were conducted using a designed system (Fig. 1a). This system comprised an ultra-sensitive surface pressure sensor (PS4, KSV NIMA, Espoo, Finland) equipped with the DyneProbe (Kibron, Helsinki, Finland), a dedicated acrylic chamber, a teflon vessel, and a device designed to simulate an electronic cigarette. The electronic cigarette simulation device was composed of commercially available components, including a 3.7 V lithium-ion battery, a micro air-pump, and a clearomizer (Kangertech CC/T2).

To initiate the experiment, an appropriate volume of poractant alfa enriched with 10% (w/w) of cholesterol (referred to as natural PSurf) was spread over a subphase of 10 mM PBS containing 0.2 mM EDTA in the teflon vessel. This step was performed using a Hamilton microsyringe until the surface pressure reached the desired initial value of 30 mN/m. The monolayer was then left undisturbed for a 20-min equilibration period prior to each measurement. To assess changes in surface pressure of the pulmonary surfactant model caused by exposure to e-cigarette vapors, measurements were conducted using three different e-liquids: a base solution (PG:VG 90:10 v/v), pure VEA, and a mixture of the base solution and VEA in a 50:50 ratio (v/v).

The vaping protocol (see Fig. 1b, c) involved five vaping sessions spaced 10 min apart. Each vaping session included 10 puffs, each lasting 3 s, with a 15-second interval between puffs. The airflow rate was 14.7 (± 0.5) mL/s. After each measurement, the system was left undisturbed for a period of 30 min to monitor any potential changes in surface pressure. Measurements were performed at ambient conditions (atmospheric pressure and room temperature) and were repeated at least three times for each e-liquid. As a control, surface pressure changes over time were also monitored for the natural PSurf without vaping throughout the duration of the experiment.

### Langmuir balance experiments

Langmuir film measurements were performed on two systems. Namely, natural lung surfactant extract, poractant alfa, enriched with 10% (w/w) of cholesterol (Chol) (referred to as natural pulmonary surfactant, natural PSurf) and a PSurf model (referred to as synthetic PSurf model). The lipid composition of the synthetic PSurf model was comprised of DPPC/POPC/POPG/Chol mixed at the molar ratio of 50/25/15/10, which corresponds very closely to the physiological concentrations of these lipids in natural pulmonary surfactant. The fluorescent probe, DOPE-Atto633, was added to the chloroform solutions of lipids and VEA (0–40 mol%) at the lipid/probe molar ratio of 1000/1. Commercially available MicroTroughXS setup (Kibron, Helsinki, Finland), equipped with an in-house modified stainless steel trough with PTFE edges and a quartz-glass window, was placed on an inverted fluorescence microscope (Olympus, Hamburg, Germany) above the water-immersion UPlanSApo 60× objective (NA 1.2, WD 0.28 mm, Olympus). The trough was filled with 10 mM PBS (containing 0.2 mM EDTA) and the natural/synthetic PSurf mixture in chloroform was deposited over the air-water interface with Hamilton microsyringe. After chloroform was allowed to evaporate and the film to equilibrate (∼ 10 min), surface pressure-surface/molecular area isotherms were collected with an ultra-sensitive surface pressure sensor (KBN 315; Kibron) with the DyneProbe. Two barriers controlled by FilmWare, the software provided by the equipment manufacturer, were symmetrically compressing the film at a constant rate of 3.92 Å^2^/mol/min. The probe was excited with a Mercury lamp while the intensity of the light was controlled with neutral density filters. Dichroic cube Cy5-A-Basic-000 (Semrock) with 630/38 and 694/44 as a single-band excitation and emission filters, respectively, was inserted in the pathway of the light that was finally detected with a CCD camera (Olympus, Tokyo, Japan). Wide-field fluorescence images were collected during the compression. The time exposure within the range of 30–75 ms was found to be optimal for recording to prevent saturation of the camera. Measurements were done at the temperature of 34.4 °C that was controlled with a temperature control plate (connected to a water-circulating thermostat; ± 0.5 °C accuracy) placed under the trough. To slow down subphase evaporation and protect from dust and additional surface disruptions, an acrylic cover box was placed over the film for the duration of a complete experiment. To balance the evaporation of the buffer in the trough, it was slowly but continuously refilled with a peristaltic pump from outside of the barriers.

### Atomic-scale molecular dynamics simulations

Atomic-scale simulations were used to find out how VEA interacts with the PSurf film and affects its physical properties at different surface pressures. PSurf was described as a lipid layer and surface pressure as the average area occupied by lipids (area per lipid, APL). The lipid composition of each monolayer system was composed of 50 mol% DPPC, 25 mol% POPC, 15 mol% POPG, and 10 mol% cholesterol^39^, hence matching the lipid compositions used in experiments of the synthetic PSurf film. The Packmol software^40^ was used to construct the systems. The values of APL used in the simulations were 50, 60, 70, 90, and 110 Å^2^, covering the APL range used in the Langmuir trough experiments. Lipid head groups were hydrated with a sufficient number of water molecules (Table 1), and potassium and chloride ions were added to represent the salt and maintain the neutrality of the simulation systems. The simulation box was implemented in such a way that it contained two opposite monolayers (having the same lipid composition), with both a thick water phase and a thick air phase between them. The head groups of lipids were towards the water and the hydrocarbon chains towards the air phase. This double-layer setup enables the air and water phases to be permanently separated from each other. In practice, the two monolayers were separated by a vacuum/air region with a thickness of at least 12 Å. The VEA (α-tocopherol acetate) molecules (20 mol%) were initially placed in the vacuum region. Table 1 provides the detailed molecular compositions of the systems.

The Slipid force field^41–44^ was used to describe lipids and α-tocopherol acetate molecules. The OPC^45^ model was applied for water^46^. The electronic continuum correction (EEC), accounting for electronic polarization, was used for ions^47,48^. The temperature of 310.15 K was maintained by the Nosé-Hoover thermostat^49,50^ with the coupling constant of 0.5 ps. The PME algorithm^51,52^ was used for handling the electrostatic interactions with a cutoff between the real and reciprocal parts of 1.4 nm.

The systems were energy minimized using the steepest descent algorithm and simulated in the NVT ensemble with the time step of 2 fs. The GROMACS 2020 simulation package^53^ was used in the simulations. Three replicas for all different APLs were built and simulated. However, due to the use of the double-layer setup, there were six practically independent replica systems. The simulation time for each simulation was set to 1000 ns, with the first 800 ns treated as the equilibration phase, during which the system reached a stable configuration. The last 200 ns of each trajectory were analyzed. The results presented in this study contain the averaged data over the different replicas.

Density profiles were calculated using the built-in GROMACS^53^ tool *‘gmx density’*. The GROMACS tool *‘gmx order’* was used to calculate the deuterium order parameters *S*_CD_. The GROMACS tool *‘gmx mindist’* was used to compute the number of contacts between different types of molecules with a default cutoff of 0.6 nm. In these calculations, VEA molecules were treated as individual entities, while the interacting molecule type was considered a single joined group. For instance, to determine the contacts between VEA and DPPC, interactions between each VEA molecule and all DPPC molecules as a group were evaluated. The resulting averages over all VEA molecules were normalized by the total number of VEA molecules in the system. Control simulations were performed utilizing lipid films without VEA.

**Table 1:** Molecular compositions of the simulated monolayer systems explored in this study

	VEA	DPPC	POPC	POPG	Chol	Water	K^+^	Cl^−^
With VEA	64	128	64	38	26	10,000	86	36
Without VEA	0	128	64	38	26	10,000	86	36

These numbers of molecules and ions correspond to two monolayers system

## Results and discussion

### Exposure to e-liquid vapor alters the surface pressure of pulmonary surfactant model

The EVALI epidemic has dramatically increased interest in understanding the physiological effects of vape additives on PSurf. Here, surface pressure changes of PSurf model upon exposure to e-cigarette vapor were monitored in real-time using the vapor exposure system designed for this work with a controlled vaping protocol (Fig. 1a–c). To model the vaping behavior of regular e-cigarette users, we used a puffing protocol in which the puff duration was 3 s and the time between puffs was 15 s. This protocol is consistent with previous studies on puffing topography among e-cigarette users^54,55^. The airflow used was 14.7 mL/s, which falls approximately in the middle of the range reported in previous studies, which varied from 8 to 39 mL/s^56^.We administered 10 puffs per session, for a total of 5 sessions, with 10 min between sessions. The chosen puffing protocol is a realistic description of the puffing behavior of e-cigarette users and enables the study of the effects of the e-cigarette vapor on PSurf model in a controlled and reproducible manner. The developed method is very sensitive, as the sensor detects surface pressure changes very precisely as highlighted by Fig. 1b, c, which demonstrate the puffing phase (recorded as a 3-second peak) and a 15-s interval. The momentary increase in pressure values is due to vibration which occurs during operation of the air pump, when the pump injects vapor into the measurement chamber.

Measurements were performed using an exogenous pulmonary surfactant model (referred to as natural PSurf). This was accomplished by adding 10% (w/w) cholesterol, which is a physiologically relevant amount, to poractant alfa, an extract of natural porcine lung surfactant used in the treatment of respiratory distress syndrome in premature infants. To evaluate the effect of VEA on PSurf model, we used the natural PSurf without VEA as a control (Fig. 1d1), and the natural PSurf exposed to three different e-liquids: a base solution (PG: VG 90:10 v/v) (Fig. 1d2), a mixture of VEA and base solution (50:50 v/v) (Fig. 1d3), and a pure VEA solution (Fig. 1d4).

The use of both PG and VG as the base solutions of e-liquids is well-established^2–4^. They act as carrier agents for nicotine and flavors, ensuring their even distribution throughout the e-liquid mixture. Moreover, the PG and VG combination allows for control over the e-liquid viscosity. When heated, VG produces dense vapor clouds while PG provides a stronger throat sensation. We selected a 90:10 ratio of PG to VG for this study because, at this composition, the production of volatile organic compounds is relatively low, reducing the likelihood of these compounds adsorbing onto surfaces and altering surface pressure^5^. However, it is important to note that in the context of health, harmful compounds such as acetone, acrolein, propionaldehyde, acetaldehyde, and formaldehyde are produced at all PG to VG ratios^3,5,57^.

As shown in Fig. 1d4, e, the surface pressure of PSurf increases by 7.43 ± 1.1 mN/m when exposed to pure VEA during the first puff session. The increase in surface pressure caused by VEA is quite radical compared to the control experiment (Fig. 1d1) where no vapor is supplied to the measurement chamber. After the first puff session, the surface pressure of PSurf decreases slightly but systematically due to slow reorganization of the PSurf film in the course of the long experiment (the surface pressure decreases by 0.34, 0.56, 0.85, 1.1, and 1.59 mN/m corresponding to times 46, 58, 70, 82, and 100 min after the start of the experiment, respectively).

Exposure to the 90:10 PG vapor mixture resulted in a significant decrease in the surface pressure of PSurf by 3.47, 5.20, 6.32, 7.31, and 8.02 mN/m after each puffing session (Fig. 1d2, e), in contrast to the effects observed with pure VEA. This decrease is likely due to the thinning of the lipid layer caused by propylene glycol and glycerin, as reported in prior studies^58,59^. Such thinning compromises the pulmonary surfactant’s ability to maintain alveolar stability, which is essential for effective lung function. Over time, repeated exposure to these vapors could contribute to chronic respiratory issues, including decreased lung compliance and an elevated risk of conditions such as chronic bronchitis or chronic obstructive pulmonary disease (COPD)^60,61^. Recent studies further support these concerns, showing that e-cigarette aerosols can induce oxidative stress, inflammatory responses, and alterations in immune function and lipid metabolism within the lungs, all of which can exacerbate the degradation of pulmonary surfactant and increase the likelihood of chronic respiratory conditions^62,63^. An additive effect of VEA and liquid base is observed in a liquid containing 50% VEA (Fig. 1d3, e). Since the surface pressure increases after the first two puffs by 3.13 and 1.2 mN/m, respectively, and then decreases after additional vapor doses by 1.02, 2.22, and 3.33 mN/m relative to the initial surface pressure before the first puff, it can be concluded that the VEA has a higher affinity for PSurf than PG and VG.

The increase in the surface pressure of PSurf may be due to reorganization of the lipid layer in a way that would force the layer to expand. The most obvious interpretation for this is that molecules from the VEA vapor are adsorbed onto the PSurf, either on its surface and/or inside the surfactant. It is known that changes in the surface tension of pulmonary surfactant can have a significant effect on lung function and thus on health^64^. Alterations in surfactant composition affect its biophysical properties, compromising its ability to maintain surface tension, which is necessary for lung function to support respiration. An increase in surface tension may impair gas exchange and trigger an inflammatory cascade in lung tissue, which is one of the putative mechanisms involved in the development of EVALI^10,11^. As a note, VEA, an α-tocopherol molecule with its hydroxyl group blocked by an acetate moiety to prevent oxidation, can still partially decompose during vaping^65^. Hence, the changes in surface pressure observed in this study may be partially attributed to the decomposition products of VEA. However, this does not alter the conclusion that the presence of VEA in the e-liquid is responsible for these effects.

For the natural PSurf model, we started with a surface pressure of 30 mN/m, which is lower than the typical pressure in healthy lung surfactant in alveoli (> 55 mN/m). Such conditions enhance our observation of how VEA interacts with natural PSurf, because adsorption kinetics are faster at lower lateral pressures of the film. Additionally, we chose this lower surface pressure to align with the conditions used in studies of the synthetic PSurf model, which cannot withstand pressures above 55 mN/m without collapsing.

### Vitamin E acetate alters the phase behavior of the pulmonary surfactant film

**Fig. 2:**
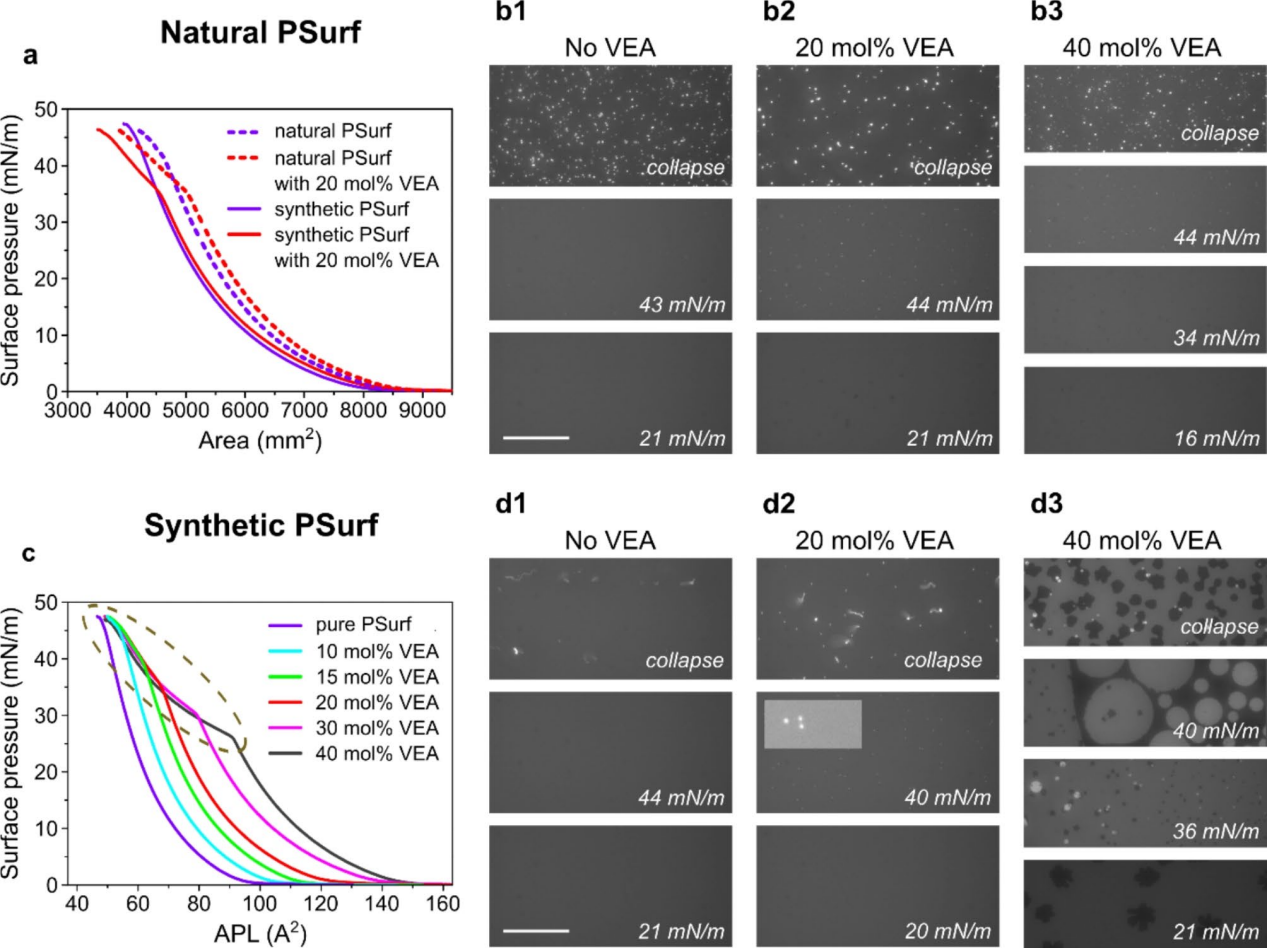
Isotherms and structures of the natural and synthetic PSurf. **a** Surface pressure-surface area isotherms of natural PSurf containing 0 and 20 mol% of VEA. Here, isotherms of synthetic PSurf with/without 20 mol% VEA are presented for comparison. **b1**–**b3** Wide-field images of natural PSurf containing 0, 20, and 40 mol% of VEA collected at three representative surface pressures. **c** Surface pressure isotherms of synthetic PSurf vs. average area per lipid (APL) containing 0 to 40 mol% of VEA. The region indicating a change in phase behavior is highlighted (dashed line). **d1**–**d3** Wide-field images of synthetic PSurf with 0, 20, and 40 mol% VEA collected at three representative surface pressures. The inset in **d2** (middle) represents a magnification (× 4) with better brightness and contrast. Scale bar is 50 μm.

VEA alters the surface pressure of the PSurf film, so it is obvious that VEA thus modifies the physical properties of the film. To investigate this, we used the Langmuir trough method, starting with a natural lung surfactant extract (poractant alfa) enriched with 10% (w/w) of cholesterol (referred to as natural PSurf). This system has been found to be a good model for the original PSurf^26^. Natural PSurf in chloroform was hence premixed with well-controlled amounts of VEA (0, 20, or 40 mol%) and deposited on the subphase drop by drop. We then measured isotherms probing the relationship between surface area and surface pressure in experiments that also produced fluorescence micrographs of mixed films at different lateral pressures. The results show that the effect of VEA on the isotherms is significant. This is illustrated in Fig. 2a, where 20 mol% VEA causes a significant change in the isotherm relative to pure natural PSurf. When the surface pressure reaches a level of ~ 32 mN/m, this change observed in the isotherm indicates that VEA induces a phase transition, thereby affecting the mechanical properties of PSurf. At higher surface pressures (32–42 mN/m), we did not observe clear differences when comparing the fluorescence images of the two systems (natural PSurf with/without VEA) (Fig. 2b). When the surface pressure exceeds the level of ~ 42 mN/m, similar inhomogeneities were observed in both cases, depicting the onset of membrane undulations.

We investigated the changes caused by VEA in more detail using a synthetic PSurf model. For this purpose, we prepared chloroform solutions of a PSurf lipid mixture (DPPC/POPC/POPG/Chol; molar ratio 50/25/15/10)^66^ enriched with varying VEA molar concentrations (0–40 mol%) and layered on top of the PBS subphase. To collect microscopy images, DOPE-Atto633 was added to the solutions at a probe/lipid molar ratio of 1/1000 before deposition. After the chloroform had evaporated and the film stabilized at the water-air interface, lateral pressure isotherms vs. APL (average area per lipid) were recorded during membrane compression. Figure 2c illustrates how an increase in VEA concentration shifts the isotherms systematically towards higher surface pressures, showing that VEA accumulates on the PSurf membrane already at low surface pressures. Even at a low VEA concentration (10 mol%), as the APL decreased, the surface pressure of the synthetic PSurf film was observed to increase in the same way as in the VEA-free composition. This indicates that in the synthetic PSurf model, a certain amount of VEA remains at the interface in the entire surface pressure range, in other words, it is not squeezed out of the PSurf film like in a collapse process.

At higher concentrations of VEA, the behavior changes. Even a concentration of 15 mol% causes a kink in the isotherm shape at a surface pressure of about ~ 38 mN/m, describing the behavior characteristic of a phase transition. The same transition can be observed in the remaining isotherms representing the other synthetic PSurf models. However, at higher VEA concentrations this change appears at lower surface pressures (Fig. 2c), based on which it would seem obvious that VEA aggregates at the air-water interface. Further, it should be noted that the isotherms corresponding to systems with VEA concentration of 15–40 mol% essentially overlap at high surface pressures over a wide range of APL (illustrated as an ellipsoid in Fig. 2c). Based on this, the lipids of the synthetic PSurf system have a limited capacity to host VEA, since squeezing out of VEA is observed upon compression.

In parallel with isotherm measurements, wide-field images of the film were collected at specific surface pressures during compression (Fig. 2d1–d3). They show that the change in the phase behavior of the synthetic PSurf correlates with inhomogeneity-like structural changes, which are observed in the fluorescence images as fine brighter structures. Their size and intensity increase slightly with lateral compression, starting from the kink observed in the isotherm and extending until collapse. Apparently, when the VEA is squeezed out of the interface, the probe molecules follow along with it, whereby the local 3D inhomogeneities of the film would manifest as observed bright structures before the collapse of the film. Interestingly, the observed structural change was most pronounced in the synthetic PSurf system at 40 mol% VEA concentration, where the phase change appeared as dark regions, while no corresponding structural change was observed in the natural PSurf film enriched with 40 mol% VEA.

The stability of the investigated systems at very high surface pressures (> 47 mN/m), corresponding to a collapse, was investigated by recording a movie of their behavior as viewed from below. Imaging showed that the subphase below the natural PSurf interface has very little freely swimming aggregates (visible as bright structures) compared to the synthetic PSurf underside where such aggregates were observed. It is reasonable to assume that the proteins in the natural lung surfactant extract (SP-B, SP-C) improve its stability, so that the PSurf material hardly escapes to the subphase even at very high surface pressure. The presence of VEA at the interface did not change the approximate concentration of aggregates in the subphase.

In summary, the experiments demonstrate that VEA modifies the surface pressure and mechanical properties of synthetic PSurf films, causing phase transitions and aggregation at various concentrations. Comparable effects were noted in natural PSurf extracts, indicating that the impact stems from interactions between VEA and lipids. Additionally, proteins in the natural PSurf extract contribute to the film’s stability, hindering the loss of material even under high surface pressure conditions.

### Vitamin E acetate clusters into aggregates and deforms the pulmonary surfactant

**Fig. 3:**
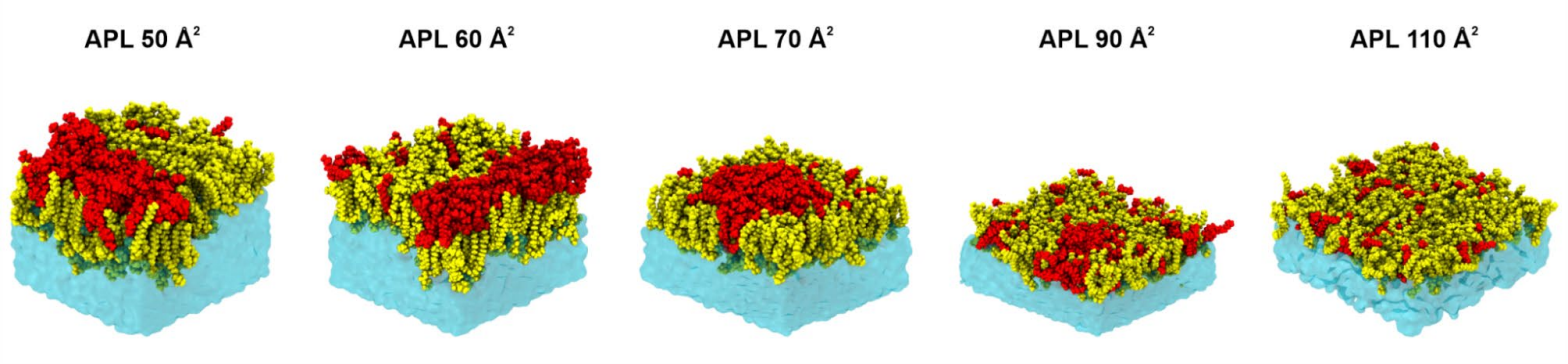
Representative snapshots of atomic-scale MD simulation systems. The simulation model has a PSurf monolayer, into which the VEA molecules originally in the air phase integrate spontaneously. The effect of surface pressure is described by varying the average area per lipid (APL), whereby a small APL corresponds to a high surface pressure (and vice versa). For clarity, only half of the simulation box is depicted. Color coding: VEA (red), PSurf lipids (DPPC, POPC, POPG, Chol) (yellow), and water (blue surface).

**Fig. 4:**
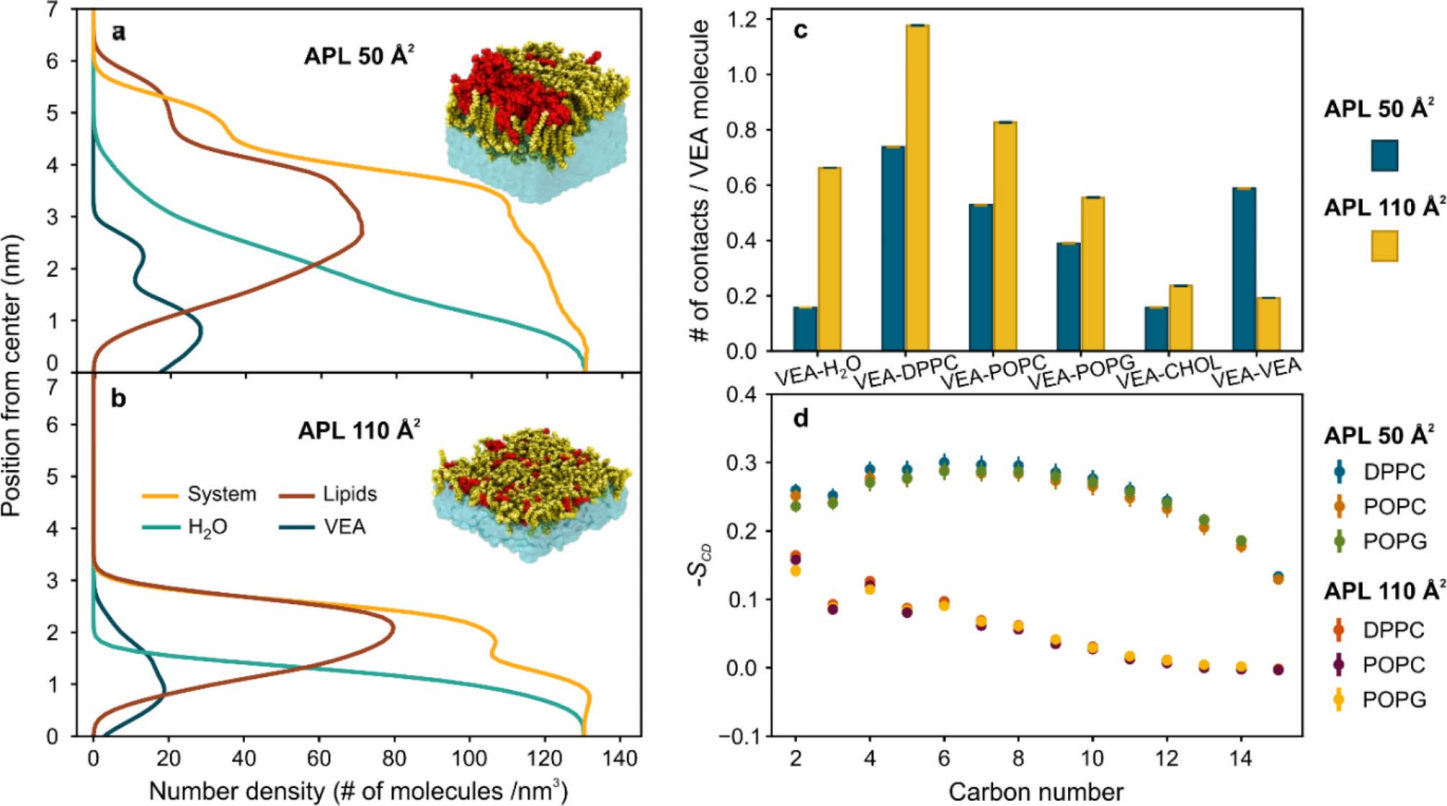
Molecular-level model of PSurf lipid film. **a** The number density profiles at the APL of 50 Å^2^ along the direction that is perpendicular to the PSurf layer plane. The center at the origin corresponds to the center of the water phase. **b** Similar number density profiles at the APL of 110 Å^2^. **c** The average number of contacts between a VEA molecule and the different lipid species for the systems with APL 50 Å^2^ and 110 Å^2^. The error bars indicate the standard error of the mean (SEM) and are about the thickness of the line. **d** The *S*_CD_ order parameters for the *sn-*1 chain of DPPC, POPC, and POPG in systems with APL 50 Å^2^ and 110 Å^2^. Error bars show the SEM.

To gain a detailed molecular-level understanding of the interactions between the PSurf and VEA molecules, we investigated this issue using atomic-scale MD simulations. The simulations used PSurf monolayers whose surface pressure was varied using the average area per lipid (APL) between 50 Å^2^ (high surface pressure) and 110 Å^2^ (low surface pressure). The VEA molecules were initially placed in the air/vacuum phase, from where they moved spontaneously to the PSurf layer during the simulations. Representative snapshots of the simulations are shown in Fig. 3, which readily depicts that the VEA molecules partition into the PSurf in all cases with their acyl chain positioned between the acyl chains of the phospholipids, while the other part of the molecule being close to the phospholipid headgroups. However, only at low surface pressure (APL 110 Å^2^) the VEA molecules mix into the membrane quite uniformly. As the surface pressure increases, the VEA molecules cluster, thus confirming the behavior interpreted from the experimental data. Interestingly, as the surface pressure increases (APL decreasing from 70 to 50 Å^2^), VEA molecules cluster to an increasing extent, especially at the interface between the lipid headgroups and water, while inducing deformations in the structure of the PSurf layers (as compared to the systems without VEA). These observations are quantified by the number density profile of VEA (Fig. 4a, b), and the number of contacts between VEA and other molecules (Fig. 4c), which clearly demonstrate the clustering tendency of VEA for increasing surface pressure (Fig. 4a, b). These predictions given by the simulations are consistent with the experimental isotherms and imaging data, showing that as the surface pressure increases, VEA aggregates and partially squeezes out of the water-PSurf interface, causing deformations to the PSurf.

No significant lateral heterogeneity was observed in the structure of PSurf, which would be due to the arrangement of lipids in different domains. This is supported by data describing the conformational order of lipid hydrocarbon chains (Fig. 4d), which is substantially identical for all lipid types.

In essence, the atomic-level simulations show that VEA is recruited with high affinity to the PSurf. As the surface pressure increases, VEA aggregates into clusters and induces deformations in the PSurf, disrupting its elastic properties, which are essential for the function of pulmonary surfactant during the respiratory cycle. In control simulations, without VEA in the film, no monolayer deformation even in the lowest APLs were observed (results not shown).

## Conclusions

The goal of this work was a comprehensive investigation and analysis of vitamin E acetate and its effect on pulmonary surfactant models, finding out the reasons for the development of EVALI. Through a combination of in vitro models and atomistic molecular dynamics simulations, we were able to showcase that VEA accumulation in the pulmonary surfactant model not only enhances its lateral surface pressure but also alters its phase behavior and mechanical properties. Moreover, the MD simulations provided unique molecular-level information of the mechanism of action of VEA, indicating that under increasing surface pressure, VEA molecules cluster into aggregates that cause deformations to the PSurf and alter its elastic properties, thereby contributing to the deterioration of lung function. This understanding is pivotal, given the dynamic nature of PSurf during the breathing cycle.

Our findings also emphasize the role of PSurf packing in the partitioning of VEA from the lung’s air phase further into its tissues. This deeper integration into the tissue may be of particular concern in this context, as VEA may alter pulmonary surfactant properties and may, after desorption, reach the subphase of the PSurf in the alveoli. Furthermore, our experimental results for pure PG and VG mixtures, given their widespread use in e-liquids, underscore the need for further research into different PG:VG ratios to fully understand their impact on lung health. Future studies should explore these ratios and include detailed experiments like those conducted for VEA, providing a more comprehensive understanding of how common e-liquid components may contribute to long-term lung damage.

It is important to note that this study utilized model systems to examine the effects of VEA on pulmonary surfactant, and this approach has inherent limitations. For example, the puffing protocol used does not fully replicate real-life vaping scenarios, where varying PG and VG ratios and different e-cigarette flow rates could result in the production of additional surface-active species. Likewise, the PSurf model employed here lacks non-lipid components and does not account for the full pulmonary interface. Furthermore, the computational modeling addressed only relatively short length and time scales of VEA-pulmonary surfactant interactions. Nevertheless, despite the individual limitations of these methods, their combined use demonstrates that VEA from e-liquids affects all pulmonary surfactant models and is unlikely to be neutral toward the natural surfactant.

Given the widespread use of various additives in the food and pharmaceutical industries, our findings underscore the importance of careful consideration when using such agents in contexts such as vaping. Although VEA is not currently used, other seemingly harmless additives in vaping products, such as flavorings, colorants, or medium chain triglycerides used as lipophilic solvents may also pose risks. The potential adverse effects of these additives, as suggested by this study, call for enhanced monitoring of their use, especially in new applications.

## Data Availability

The data that support the findings of this study are available from the corresponding author upon reasonable request.
